# A Remedy for the Great Pustulous Eruption, and Its Degrees

**Published:** 1799-10

**Authors:** William Owen

**Affiliations:** Penton-Street, Pentonville


### *To the Editors of the Medical and Physical Journal.*

Gentlemen,

In looking over old Welsh manuscripts for selecting materials for the Welsh Archaiology now printing, I casually met with the following fragment, which, as it relates to the history of the venereal disease, I have sent a translation of it to you, under the idea it might be an acceptable article in your valuable publication.

I remain, Gentlemen,

Penton-Street, Pentonville, Your's, &c.

*Sept.* 9, 1799. WILLIAM OWEN.

### *A Remedy for the Great Pustulous Eruption, and its Degrees.*

This remedy was sent by M. Ry. Tiler, a French physician, when it was the year of our Lord, one thousand and five hundred, save six years, to King Henry the Seventh, the first person in this kingdom who was afflicted with that disease. It was turned into English by Mr. Stradling; and Davydd ab Meirig Ddu turned it into Welsh.

There

There are nine ſorts of diſorders of the great puſtules; and there are five of them irremediable, and not to be helped; and there are four againſt which there is a certain remedy: and, of the nine, there never came but three into this iſland, and people have two of them frequently; but the third, let it be guarded againſt, which is called in France *mabtai yſums yſvn*; that is, the dry eruption, the baſis of which proceeds from the heart.

The firſt of the three is of a cold and dry nature. Its ſymptoms are ſhivering and chillineſs, and nevertheleſs ſweaty; pain in the ſhoulders, or in the other joints, from the loins upwards, and yet full of fleſh, and craving for ſweet things. In the height of the diſorder, a kind of dry heads break out, with black eyes in them, and void of matter, growing bigger and bigger, from nipples to teats, like dry warts, in the end growing large and in irregular lumps, breaking into narrow wounds.

THIS IS THE REMEDY.

If the perſon is not freed from the pain in his joints, let him have this emetic: take a quantity of the bark of the walnut-tree *, throwing away the upper rind, then bruiſe it moderately ſmall, and waſh it in clean water; then take a quart of Rheniſh wine, or Malmſey, or old ale, and put the bark into it to ſtand for three hours, ſo that it becomes viſcid, then ſtrain it clean, and put it on the fire to be warmed a little, and when warm, throw into it three-pennyworth of long pepper. Put the patient in bed until nine o'clock, and then give him the above drink, and at eight at night, and the ſame time next morning, and he will diſcharge a cruel quantity of obnoxious matter and impurity. Take the weight of ij. drachms of ſpermaceti, and the weight of iiij. of pepper in powder, and throw it upon wine, or old ale, gently warmed, and give it to the patient to drink, and there will be an eruption of what lurks in the body.

Guard againſt applying too much ointment; for the three evils attending the cure are, the extinction of the veins, ſending away of the blood, and filling them with poiſon; for it is dry and heating; on that account, better is an emetic with the unction, for the ſtrength of the perſon.

The ſalve for the pain of that pox is this: Take the leaf of fat of a red pig, and take away the membrane; take two parts of it, and pound it in a mortar well, then take a pennyworth of quickſilver, and kill it well: this is the way it is to be killed; take ſome urine in a cup and throw the quickſilver into it, and ſtir it with the finger, until it is ſeen to ſeparate into particles like the heads of ſmall pins, which are to be thrown into the mortar with

* I am not certain whether the walnut or filbert-tree is meant, from the name given it here.

with the lard, and the whole to be beaten well together, until it appears blue, from the colour of the quickfilver. Then take the weight of iij. drachms of maftic, and pound it into fine powder, and take the weight of ij. drachms of fpermaceti, and put thefe two things with what is above-mentioned, and pound them well a fecond time. Then take the third part of lard before referved, and put in a pan on the fire to be melted with ij. drachms of camphire in it; and then pour what is melted into the mortar to the above ingredients, and let the whole be again well pounded and mixed in that manner. Take of the oil of bays two ounces, and two ounces of Exeter oil, and pound them alfo with the above in the mortar, until you fee them of the colour of the Exeter oil, and then put the ointment in a box or other clean veffel, to be well kept. Put the fick perfon in bed, with fufficiency of cloaths on him to caufe a gentle fweat. . . . . . . .

[*Here it breaks off, owing to there being leaves loft in the manufcript.*]

W. O.

☞ We have inferted the above fragment, to fhew the firft preparations of mercury in the cure of lues.—Editors.

